# Hand hygiene intervention to optimise soil-transmitted helminth infection control among primary school children: the Mikono Safi cluster randomised controlled trial in northwestern Tanzania

**DOI:** 10.1186/s12916-021-01987-6

**Published:** 2021-05-21

**Authors:** Kenneth Makata, Jeroen Ensink, Philip Ayieko, Christian Hansen, Simon Sichalwe, Julius Mngara, Onike Mcharo, Humphrey Mazigo, Jeremiah Seni, Robert Dreibelbis, Sarah Rockowitz, Elialilia Okello, Heiner Grosskurth, Safari Kinunghi, Saidi Kapiga

**Affiliations:** 1 Mwanza Intervention Trials Unit (MITU), Mwanza, Tanzania; 2 London School of Hygiene and Tropical Medicine, London, UK; 3 National Institute for Medical Research (NIMR), Mwanza, Tanzania; 4 Department of Medical Parasitology and Entomology, Catholic University of Health and Allied Sciences (CUHAS), Mwanza, Tanzania; 5 Department of Microbiology and Immunology, Catholic University of Health and Allied Sciences (CUHAS), Mwanza, Tanzania; 6 Johns Hopkins Bloomberg School of Public Health, Baltimore, Maryland USA

**Keywords:** Soil-transmitted helminth, *Ascaris lumbricoides*, *Trichuris trichiura*, Mass drug administration, Deworming, Hand hygiene, Handwashing, School children, Cluster randomised trial, Tanzania

## Abstract

**Background:**

Soil-transmitted helminth (STH) infections are highly prevalent in resource-limited countries. We assessed the effect of a combination intervention aiming to enhance handwashing with soap on STH reinfection following mass drug administration among primary school children in Kagera region, Northwestern Tanzania.

**Methods:**

We conducted a cluster randomised trial in sixteen primary schools with known high STH prevalence. Schools were randomly assigned in a 1:1 ratio to either receive the intervention or continue with routine health education. The intervention included teacher-led classroom teaching, parental engagement sessions, environmental modifications and improved handwashing stations. The evaluation involved two cross-sectional surveys in a representative sample of students, with the end-line survey conducted 12 months after the baseline survey. The primary outcome was the combined prevalence of *Ascaris lumbricoides* and *Trichuris trichiura* infections at the end-line survey. Secondary outcomes included reported handwashing behaviour, the prevalence and intensity of individual STHs, and hand contamination with STH ova and coliform bacteria. End-line STH prevalence and intensity were adjusted for baseline differences of potential confounders.

**Results:**

At the end-line survey, 3081 school children (1566 from intervention schools and 1515 from control schools) provided interview data and stool specimens. More school children in the intervention group reported the use of water and soap during handwashing compared to school children in the control group (58% vs. 35%; aOR=1.76, 95%CI 1.282.43, *p*=0.001). The combined prevalence of *A. lumbricoides* and *T. trichiura* infections was 39% in both trial arms (aOR = 1.19; 95%CI 0.741.91). The prevalence of *A. lumbricoides* was 15% in the intervention and 17% in the control arm (aOR =1.24, 95%CI 0.592.59) and that of *T. trichiura* was 31% in both arms (aOR=1.17, 95%CI 0.731.88). No significant differences were found for STH infection intensity in both the main study and the hand contamination sub-study.

**Conclusions:**

The intervention was effective in increasing reported handwashing behaviour at school, but failed to show a similar effect in the home. The intervention had no effect on STH infection, possibly due to infection in the home environment, other transmission routes such as contaminated water or food or limited changes in school childrens handwashing behaviour.

**Trial registration:**

The trial was registered on June 21, 2017, by the International Standard Randomised Controlled Trial Number (ISRCTN45013173).

## Background

Soil-transmitted helminth (STH) infections are a major global health problem, with more than one billion people estimated to be affected worldwide [1]. The infections are particularly frequent among school children in low- and middle-income countries (LMICs) in whom they are associated with anaemia and impaired physical and cognitive development [2, 3]. Deworming by using anti-helminthic drugs as part of regular mass drug administration (MDA) is advocated by WHO as a strategy for STH control in high prevalence areas in LMICs [1]. In Tanzania, annual MDA campaigns are conducted in primary schools as part of the national neglected tropical diseases (NTD) control programme [4]. However, STH prevalence remains high in many communities [5, 6] as MDA is often followed by rapid re-infection [7]. Poor water, sanitation and hygiene (WASH) practices have been proposed as a likely explanation [8].

The role of handwashing with soap in preventing STH transmission is yet to be established given inconsistent results from recent studies. A systematic review and meta-analysis reported in 2014 suggested that hand hygiene or other individual WASH interventions may reduce the odds of STH re-infection following deworming by 3370% [9]. However, a more recent review reported inconsistent findings, with hand hygiene effectiveness ranging from zero to 59% [10]. None of the studies included in this review assessed the effect of handwashing with soap alone as a single intervention on STH re-infection after treatment.

We conducted a cluster randomised trial to assess the effect of a combination intervention aiming to enhance handwashing with soap on STH re-infection following MDA among primary school children in Kagera region, northwestern Tanzania. This region has high prevalence of *Ascaris lumbricoides* and *Trichuris trichiura* infections, which are species of STH known to be transmitted predominantly through the oral ingestion of worm eggs [5, 6].

## Methods

### Study setting and population

Kagera region is situated on the western shores of Lake Victoria, neighbouring Rwanda and Burundi to the West and Uganda to the North. Based on the national census, Kagera had a population of about 2.5 million by 2012, with an annual inter-census growth rate of 3.2% [11]. The primary school system is mainly public, with high rate of school enrolment especially in urban areas, ranging from 85 to 90% [12]. All major villages of the region have at least one public primary school which comprises of grades 1 to 7, with the number of students aged 612 years ranging from 500 to 1500 children per school.

The trial was conducted in 16 public primary schools purposely selected from a total of 51 schools assessed prior to study initiation to establish eligibility. The schools were located in 3 out of 8 districts in the region (Bukoba urban, Bukoba rural and Muleba) which were chosen because they were easily accessible from the project office in Bukoba town. The schools were selected based on pre-determined criteria: a pre-trial prevalence of *Ascaris lumbricoides* and/or *Trichuris trichiura* infections of at least 20%, access to water within the school premises, and for logistical reasons a school size of less than 1200 students.

### Study design and data collection

**Fig. 1:**
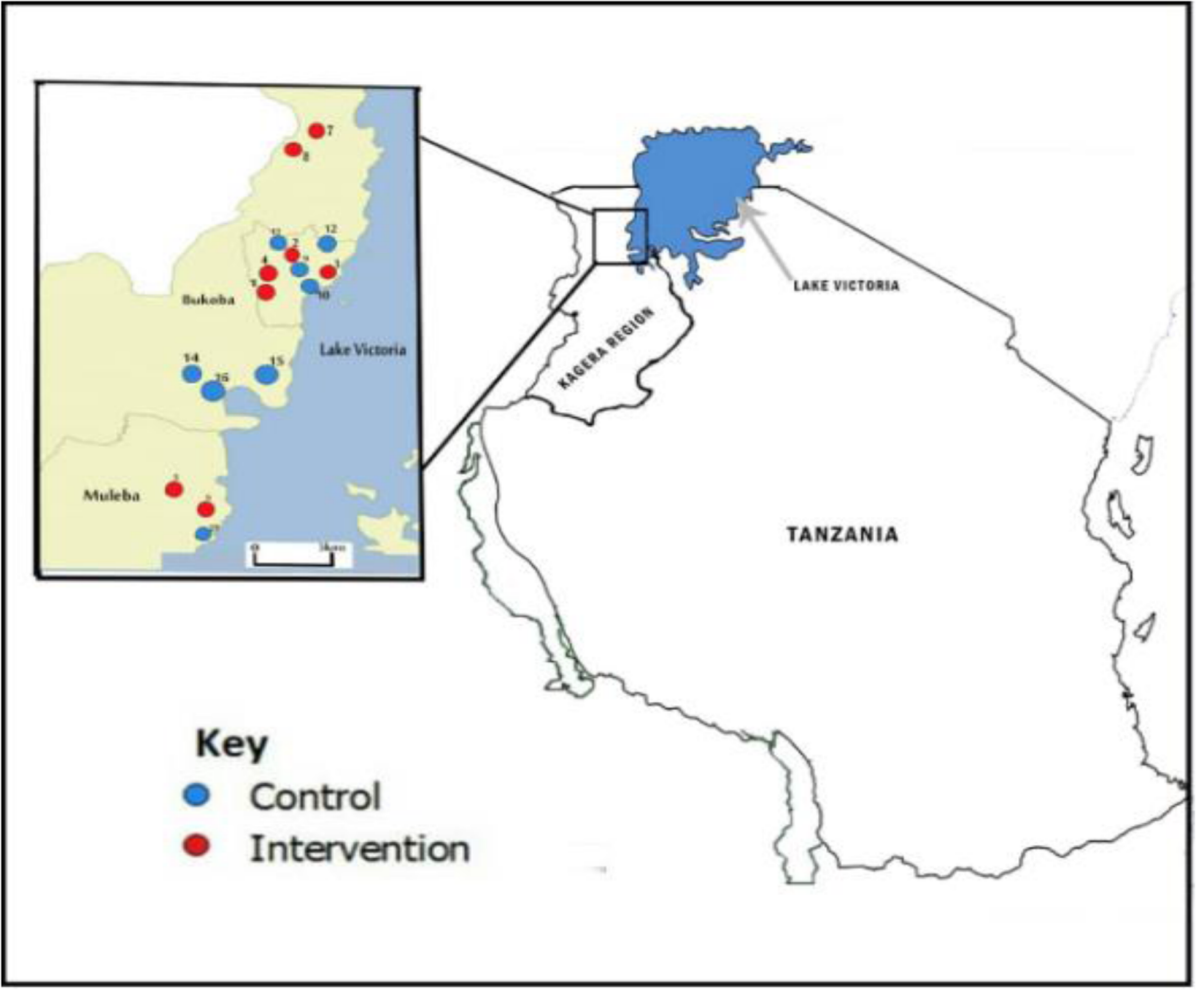
Geographical location and randomisation status of the participating primary schools in Kagera region, Tanzania

This was a cluster-randomised controlled trial (c-RCT) using primary schools as randomisation units. Eight schools each were allocated to the intervention and control arms (Fig.1). Randomisation was restricted to ensure a balanced distribution of schools across the two trial arms with respect to district location and level of pre-trial STH prevalence. Details on the study design and randomisation process have been described previously [13].

### Study objective and outcomes

The study aimed to determine the effectiveness of the Mikono Safi handwashing intervention on sustaining the prevalence of STH infections observed after performing routine deworming as part of the national MDA programme*.* Mikono Safi means clean hands in Kiswahili, the national language spoken in Tanzania. The primary outcome was defined as the combined prevalence of *Ascaris lumbricoides* and *Trichuris trichiura* infection at the end-line survey conducted 12 months after the intervention had been introduced in the intervention schools. Secondary outcomes included the prevalence and infection intensity of each STH infection, the prevalence of hand contamination with STH ova, *Escherichia coli* and other coliform bacteria, and the prevalence of reported handwashing behaviour.

### Intervention components

**Fig. 2:**
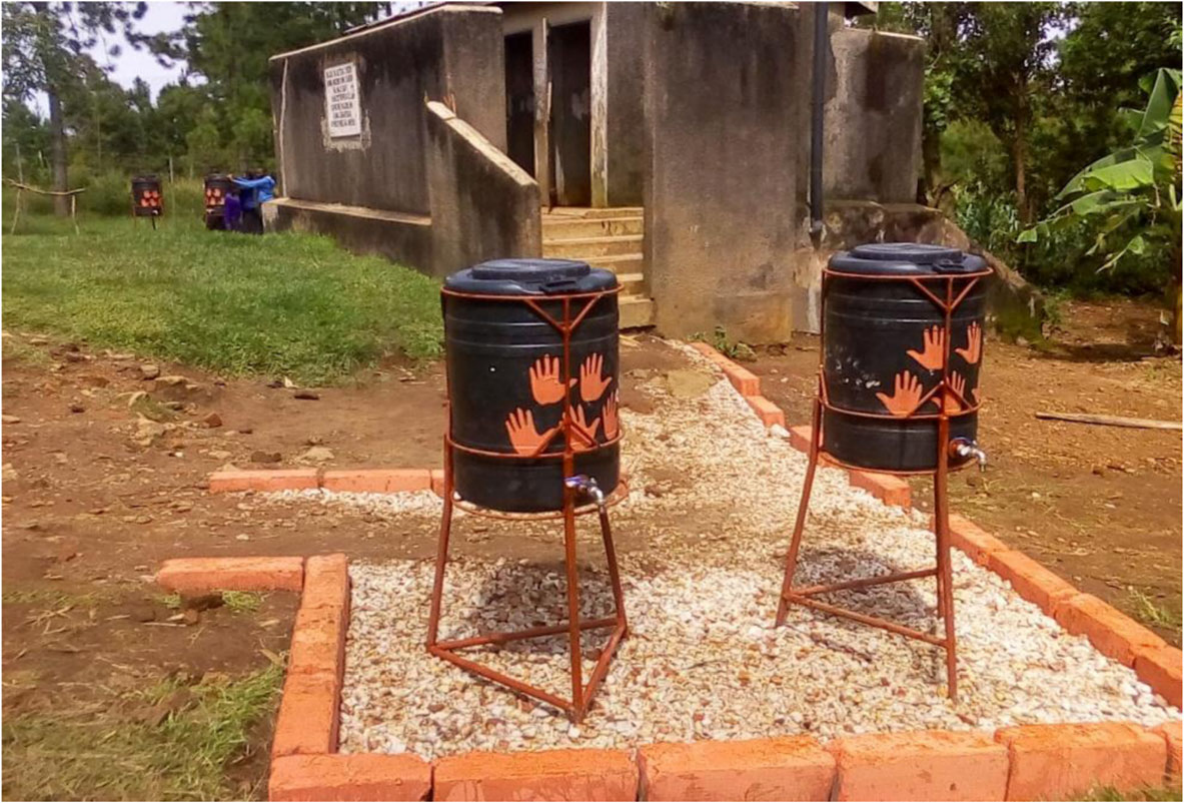
Path between toilets and hand washing facility with nudges

Details of the intervention and its implementation have been described elsewhere [13]. Briefly, the intervention comprised 3 components: health education of children to promote handwashing with water and soap, a one-off engagement meeting with parents at school to obtain their support and modest modification of the physical environment at schools to facilitate handwashing. Health education was delivered using specifically designed teaching materials in three teacher-led sessions given during the course of 1 year. The sessions combined classroom lessons and handwashing demonstrations and games. We aimed to increase parents emotional engagement and support by sharing and discussing pre-trial stool test results on STH infections of their own children. For this exercise, stool samples were collected and rapidly analysed under field laboratory conditions using the Kato-Katz method. At schools, user friendly handwashing facilities were installed in close proximity to schools latrine buildings [14]. We also marked the paths linking latrines with the near-by handwashing stations and painted brightly coloured nudges on the water containers to sub-consciously influence students handwashing behaviour [15, 16] (Fig.2). The design of the intervention had been informed by formative research conducted prior to the trial [17]. Schools in the control arm had access to water for handwashing and continued with routine health education as per national curriculum.

### Design of the evaluation

In each school, two cross-sectional surveys were conducted, a baseline survey from November 2017 to June 2018 and an end-line survey 12 months later. In each survey, we aimed to enrol 1680 students from each trial arm, an average of 210 students from each school, stratified by sex and grade. Separate independent samples of students were enrolled at baseline and end-line surveys. Interviewer administered questionnaires were used to collect information on socio-demographic characteristics, handwashing practices while at home and school, sources of drinking water, water treatment and food preservation practices, type of household latrine and other risk factors for STH infection in the home environment. Data from both surveys were recorded directly onto tablet computers with in-built checks to minimise errors. Data were uploaded daily to a secure database and checked by the data manager.

In all study schools, a school-wide MDA was conducted using a single oral dose of 400 mg of albendazole in line with the national NTD programme guidelines [4]. Two weeks later, we conducted the baseline survey at which randomly selected students were requested to provide a stool specimen for the detection of STH ova. Re-treatment was provided to all students who were still found infected on this occasion. This approach was chosen in keeping with the study protocol [13] to help determine the level of STH infections remaining at the point when the intervention package was about to be implemented and ensure that infections detected at the end-line survey were likely due to reinfections. This allowed the impact of the intervention on sustaining the effects of deworming to be established by comparing the prevalence of infections in the two trial arms at end-line survey. This approach also provided data which was used to adjust for potential baseline imbalances in remaining infections in the analysis.

In addition, a sub-study was conducted to assess levels of hand contamination with faecal bacteria and STH ova in a randomly selected 20% sample of students who took part in the end-line survey. A total of 672 students were enrolled, 336 from each arm stratified by sex and grade, about 40 per school. Students were asked to wash and brush their hands and nails within a sterile polythene bag (Biodegradable Falcon zipper bags, Falconpack, U.A.E) containing 100 ml of isotonic saline. These hand rinse water samples were collected after students had spent about 2 h attending routine activities in school. The samples were sent to the nearby Bukoba regional referral hospitals laboratory for the detection of helminth ova, faecal coliforms and *Escherichia coli*.

### Laboratory methods

Stool specimens were preserved in 10% formalin at room temperature in sealed containers and transported to the central laboratory in Mwanza for analysis. Specimens were analysed using the formol-ether concentration method to identify helminth ova as this approach has shown high sensitivity even in populations with low infection intensity as could be expected in schools participating in annual deworming [18]. Infection intensity was assessed microscopically. Briefly, 1 g of stool sample was placed in a mortar and 10ml of 10% formal saline was added. The stool sample was homogenised by grinding. The homogeneous solution was filtered using a funnel with gauze into a test tube (10ml) and 2ml of ether was added. The resultant solution was mixed vigorously and then centrifuged at 3000 rpm for 5 min. After centrifugation, the floating debris was removed using an applicator stick, the supernatant discarded to remain with the sediment at the bottom of the test tube. The sediment was examined under light microscope (first at 10 magnification and then at 40 magnification) by taking one drop of sediment at a time, placing on a microscopic slide and covering by a cover slip. All helminth ova were examined and counted until the whole sediment was completed.

For safety reasons, diethyl ether solvent was replaced by ethyl-acetate, which has been shown not to affect the results [19]. Each specimen was examined by two independent qualified laboratory technologists. Specimens with discrepant results were reviewed with consultation among the two by a third reader. Quality control was performed on 10% of randomly selected samples and a repeated examination was performed by the same technologists without knowledge of their initial results.

For bacteriological analysis, 1 mL from each hand rinse water sample was taken using a sterile Pasteur pipette and placed into 9 mL of Brain Heart Infusion Broth (BHI; HI Media, India). Samples were processed in the laboratory within 23 h after collection by inoculating them onto MacConkey agar w/0.15% bile salt, CV and NaCl (HI Media, India) using a calibrated 1 L loop. The plates were incubated at 35C to 37C for 18 to 24 h. The absolute numbers of colonies detected on the MacConkey agar plates (both lactose and non-lactose fermenting colonies) were multiplied by 1000 to get the corresponding number of coliforms CFU/ml. The resulting value was also multiplied by 10 taking in account the 1:10 sample to BHI dilution to get the final total coliforms CFU/ml. *Escherichia coli* was confirmed using conventional biochemical identification tests, and the total *Escherichia coli* CFU/ml was enumerated [20] and categorised into mild (9 10^3^ CFU/ml), moderate (1099 10^3^ CFU/ml) and high (100 10^3^ CFU/ml) intensities.

For the identification and quantification of helminth ova, 10 ml of the main hand rinse water sample was processed using the zinc sulfate centrifugal flotation method and examined by light microscopy for presence of STH eggs [21]. Helminth ova observed were quantified as described above.

### Statistical analysis

Sample size calculations were informed by data from the pilot survey which showed that combined prevalence of *Ascaris lumbricoides* and *Trichuris trichiura* infection was 30% in pilot schools with a between-school coefficient of variation (k) of 0.3. The calculations assumed that 1 year after the deworming campaign the combined prevalence of *Ascaris lumbricoides* and *Trichuris trichiura* infection in the control arm would have reached the original level of 30% seen during the pilot conducted in the absence of a handwashing intervention. We determined that with a between-school coefficient of variation of 0.3, a total of 3200 participants across 16 primary schools (~200 participants per school), would provide at least 80% and 95% power to show intervention effects of 40% and 50% relative reduction in combined helminth infection, respectively.

Data analysis was performed using STATA version 14.2, following a pre-specified analysis plan, by analysts who were blind to the trial group allocation. Descriptive analysis of the characteristics of clusters and individual participants was conducted for each trial arm and overall. The medians and interquartile ranges were calculated for participants age and reported as a continuous variable while frequency counts (percent) were calculated for categorical variables.

The primary analysis was conducted following the intention-to-treat (ITT) principle, with participants analysed according to the trial arm to which they were randomly allocated. All participants who were interviewed and provided a stool sample during the end-line survey were included in the analysis. The effect of the intervention on the primary outcome, i.e., the combined prevalence of *Ascaris lumbricoides* and *Trichuris trichiura* infection, was analysed in two stages. First a cluster level summary of the primary outcome was calculated for each school, and then, the means of the cluster summaries were compared between trial arms using an independent sample *t* test [22]. For the primary outcome, the cluster level summary represented the residuals of adjusted log odds of infection obtained from logistic regression with *Ascaris lumbricoides* and *Trichuris trichiura* infection as outcome and adjusting for covariates measured at baseline that were possibly associated with STH infection at the end-line survey. Two-sided *p* values and 95% confidence intervals (95% CI) were computed from the *t* test comparison of the mean adjusted log odds residuals of infection. The corresponding adjusted odds ratios (95% CI) were obtained using exponentiation. This two-stage analysis was repeated for categorical secondary outcomes and the categorical outcomes in the hand contamination sub-study.

We used the quantitative egg count variable rather than the categorical variable (low, moderate, high) for assessment of the impact of the intervention on the intensity of helminth infection. The effect of the intervention on this quantitative outcome and on bacterial count (in the hand contamination sub-study) was assessed using a log-linear model, assuming a negative binomial distribution with a log link in the first stage followed by *t* test comparison of mean residuals in the second stage. The corresponding adjusted rate ratio (and 95% confidence interval) for quantitative outcomes was obtained by exponentiation of the mean differences from the *t* test.

## Results

**Fig. 3:**
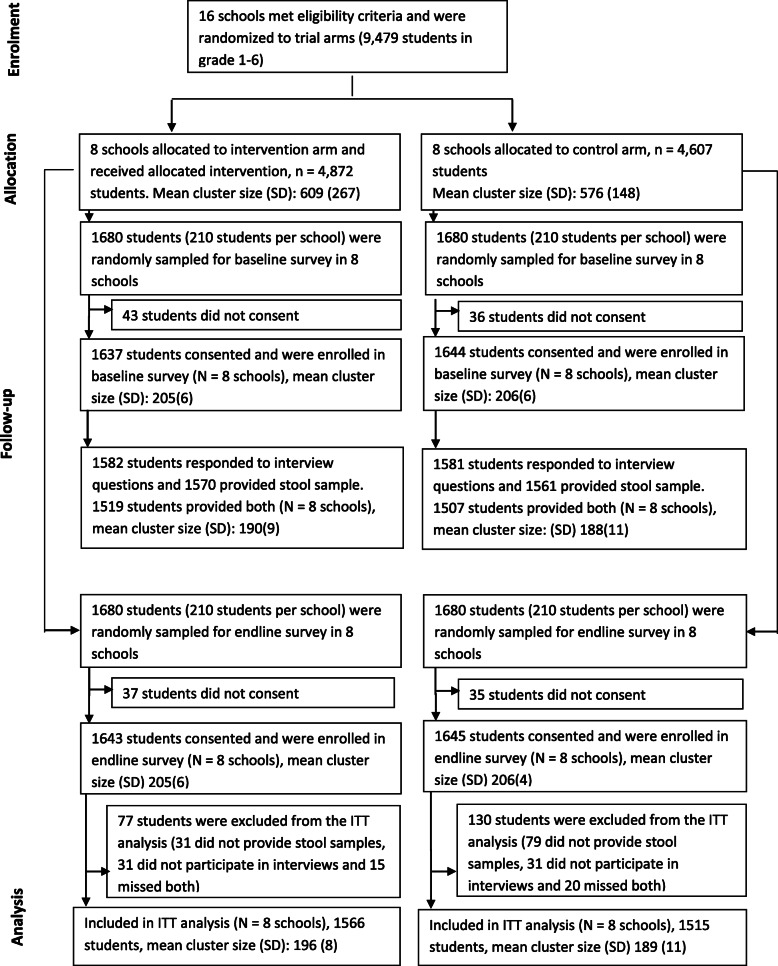
CONSORT flow diagram for Mikono Safi study

The individual follow-up time per school was about 12 months. Sixteen eligible primary schools were randomised to the intervention arm (8 schools, total number of school children=4872) or the control arm (8 schools, total number of school children=4607) (Table1). During the baseline survey, 3026 school children participated in the interviews and contributed stool specimens (1519 from the intervention arm and 1507 from the control arm). During the end-line survey, 1566 out of 1680 (93.2%) school children in the intervention arm and 1515 out of 1680 (90.2%) school children in the control arm contributed both interview data and stool samples and were included in the ITT analysis (Fig.3).

**Table 1:** Characteristics of clusters randomised to the intervention or control arm of the Mikono Safi trial, Kagera region, Tanzania

	Intervention	Control	Total
Number of participating clusters (schools)	8	8	16
Total number of children enrolled in participating schools	4872	4607	9479
Mean number of children per school	609	576	592
Number of schools located in:			
Bukoba Municipality^a^	4	4	8
Bukoba rural	2	3	5
Muleba	2	1	3
Source of school water supply			
Number of school supplied with piped water	5	3	8
Number of schools using rain water	3	5	8

^a^One-half of the schools in the trial and in each arm were located in the urban municipality and the remaining half in rural area

As expected due to the study design, the number of participants was balanced with regard to gender and students age during both baseline and follow-up surveys (Table2). The combined prevalence of *Ascaris lumbricoides* and *Trichuris trichiura* was 35% in both trial arms, determined 2 weeks following deworming campaigns. The prevalence of *Ascaris lumbricoides* alone was 0.4% and 3.1% in the intervention and control arms, respectively, while for *Trichuris trichiura*, it was 35% and 34%, respectively. Some imbalances were noted at baseline with regard to factors that could influence the risk of STH infection, but the direction of these differences did not consistently favour one of the trial arms: fewer children in the control arm reported handwashing after using the toilet (37% vs. 71%), households access to piped water was more frequent in the control arm (35% vs 25%), and more children in the control arm reported handwashing before eating (58% vs. 38%).

**Table 2:** Characteristics of individual participants within the clusters randomised to the intervention or control arm of the Mikono Safi trial, Kagera region, Tanzania

	Intervention ***n*** (%)	Control ***n*** (%)	Total ***n*** (%)
**Baseline survey**			
Number of participants at baseline	1582	1581	3163
Median [IQR] age of participants in years	10 (812)	10 (812)	10 (812)
Female sex	807 (51)	809 (51)	1616 (51)
Household possessions			
Farm/land	1500 (96)	1346 (89)	2846 (92)
Cow(s)/pig(s)/goat(s)/sheep	805 (51)	774 (51)	1579 (51)
Television	957 (61)	1010 (67)	1967 (64)
Gas/electric cooker	586 (37)	743 (49)	1329 (43)
Mobile phone(s)	1508 (96)	1461 (96)	2969 (96)
Bicycle	482 (31)	516 (34)	998 (32)
Motor cycle	280 (18)	385 (25)	665 (22)
Vehicle/tractor/boat	379 (24)	356 (23)	735 (24)
Main source of water at home			
In-house piped water	166 (10)	282 (18)	448 (14)
Public owned piped water	239 (15)	264 (17)	503 (16)
Well	99 (6)	117 (7)	216 (7)
River/stream	1040 (66)	920 (58)	1960 (62)
Lake	51 (3)	10 (1)	61 (2)
Water vendor	41 (3)	38 (2)	79 (3)
Other sources	47 (3)	38 (2)	85 (3)
Reason for handwashing the last time participant washed hands			
Had visited the toilet	1119 (71)	583 (37)	1702 (54)
Washed before eating	608 (38)	918 (58)	1526 (48)
Participant reported ever eating soil	523 (33)	575 (36)	1098 (35)
Soil-transmitted helminth infection at baseline			
Ascaris infection	6 (0.4)	49 (3.1)	55 (1.8)
Trichuris infection	543 (35)	529 (34)	1072 (34)
Hookworm infection	7 (0.45)	12 (0.77)	19 (0.61)
**Endline survey**			
Number of participants at end line	1566	1515	3081
Median [IQR] age of participants in years	10 (8-12)	10 (812)	10 (812)
Female sex	791 (51)	786 (52)	1577 (51)
Participant currently lives with			
Both parents	1024 (65)	932 (62)	1956 (63)
Single parent	268 (17)	294 (19)	562 (18)
Guardian	274 (17)	289 (19)	563 (18)
Participants who received worm treatment during the deworming campaign at start of the study	1457 (93)	1328 (88)	2785 (90)
Participant ever received worm treatment somewhere else rather than at school	390 (25)	421 (28)	811 (26)
Participants who had a latrine at home	1559 (99.6)	1512 (99.8)	3071 (99.7)
Type of latrine			
Latrine uses flushed water	531 (34)	519 (34)	1050 (34)
Latrine has a hole in the floor	1231 (79)	1158 (76)	2389 (78)
Latrine has a hole in the floor and a lid to cover the hole	421 (27)	424 (28)	845 (28)
Latrine with a ventilation pipe	566 (36)	562 (37)	1128 (37)
Reported diarrhoea episode over the past 7 days	373 (24)	337 (22)	710 (23)
Participant reported that they had ever eaten soil	622 (40)	602 (40)	1224 (40)
Participant reported that they had ever observed worms while passing stool	728 (46)	745 (49)	1473 (48)

End-line survey results largely mirrored those from the baseline survey (Table2). The majority of the school children lived with both parents (65% and 62% in intervention and control groups, respectively). The majority of parents worked as farmers or were engaged in small scale businesses. Almost all participants reported to have a latrine at home and most of these (78%) were pit latrines. Having had at least one episode of diarrhoea during the past 7 days preceding the interview was reported by about 23% of the children. About 40% recalled that they had ever eaten soil and about 48% reported to have ever observed worms during defecation.

In Table3, we present the effect of the handwashing intervention on STH prevalence, infection intensity and reported handwashing behaviour during the end-line survey. The proportion of participants who reported to have used water and soap when they last washed their hands was 58% in the intervention arm and 35% in the control arm (aOR=1.76, 95%CI 1.282.43, *p*=0.001) (Table3). When students were asked about the last time they washed hands at school, the use of water and soap was reported by 72% of children in the intervention arm and 39% in the control arm (aOR=3.7, 95%CI 1.3510.15, *p*=0.002). When asked about the last time they washed hands at home, 45% of children in the intervention arm and 33% in the control arm reported having used water and soap (aOR=1.02, 95%CI 0.741.39, *p*=0.90).

**Table 3:** Effect of hand washing intervention on soil-transmitted helminth prevalence, infection intensity and handwashing behaviour at end-line survey in the Mikono Safi trial, Kagera region, Tanzania

	Intervention (***N*** = 1566)	Control (***N*** = 1515)	Crude OR^a^ (95% CI)	aOR^ab^ (95% CI)	***P*** value
	***n***(%)	***n***(%)			
**Primary outcome**^b^					
Combined *Ascaris lumbricoides* and/or *Trichuris trichiura* infection	603 (39)	585 (39)	0.99 (0.601.63)	1.19 (0.741.91)	0.466
*Ascaris lumbricoides* infection prevalence	242 (15)	259 (17)	0.90 (0.392.09)	1.24 (0.592.59)	0.547
*Trichuris trichiura* infection prevalence	479 (31)	464 (31)	0.99 (0.601.64)	1.17 (0.731.88)	0.501
**Secondary outcomes**^c^					
Hookworm infection	11 (1)	13 (1)	-	-	-
Participants reporting using soap and water during last hand washing occasion	910 (58)	529 (35)	1.68 (1.182.39)	1.76 (1.282.43)	0.001
Reported location of last handwashing episode					
School	746 (48)	393 (26)	1.84 (1.133.00)	1.81 (1.122.93)	0.011
Home/did not remember location/other location	820 (52)	1122 (74)	1.0	1.0	
Participants used soap and water during most recent hand washing occasion in school **[*n*/*N*(%)]**	538/746 (72)	154/393 (39)	3.42 (1.279.22)	3.7 (1.3510.15)	0.002
Participants used soap and water during most recent hand washing occasion at home **[*n*/*N*(%)]**	372/820 (45)	375/1122 (33)	0.97 (0.691.37)	1.02 (0.741.39)	0.904

^a^Unless otherwise stated, figures represent odds ratios; ^b^adjusted for school baseline prevalence of *A. lumbricoides* infection, households access to piped water, hand washing with soap before eating and after using the toilet and soil eating behaviour; ^c^adjusted for school aggregated households access to piped water at baseline only

For the primary outcome measure at project end-line, the combined prevalence of *Ascaris lumbricoides* and *Trichuris trichiura* infection was 39% in both trial arms (aOR = 1.19; 95%CI 0.741.91) (Table3). The prevalence of *Ascaris lumbricoides* infection alone was 15% in the intervention arm and 17% in the control arm (aOR =1.24, 95%CI 0.592.59). The prevalence of *Trichuris trichiura* infection was 31% in both trial arms (aOR=1.17, 95%CI 0.731.88). There were no significant differences in the mean egg count between trial arms. For *Ascaris lumbricoides,* the cluster-level mean egg count was 150 eggs/gram ( 105) in the intervention arm and 305 eggs/gram ( 350) in the control arm (aOR 0.84 0.292.37), respectively. For *Trichuris trichiura*, these data were 16 ( 6) and 34 ( 19) eggs/gram, respectively (aOR 0.96; 95%CI 0.531.76). Only 1% of participants had evidence of hookworm infection, with no difference between trial arms.

A total of 672 school children participated in the sub-study on hand contamination, 336 from each arm (Table4). None of the children were found to have their hands contaminated with eggs of *Trichuris trichiura* infection, while 6% and 10% of students from the intervention and control arms, respectively, had their hands contaminated with eggs of *Ascaris lumbricoides* (OR=0.84, 95%CI 0.391.81). Few children had evidence of contamination with hookworm eggs, without significant difference between trial arms. We also did not observe significant differences between trial arms on the level of hand contamination with both coliform bacteria and *Escherichia coli*. There was a trend towards a lower intensity of contamination with regards to both coliform bacteria and *Escherichia coli* in the intervention arm; however, none of these differences were statistically significant.

**Table 4:** Effect of handwashing intervention on hand contamination with soil-transmitted helminths and coliform bacteria in the Mikono Safi trial, Kagera region, Tanzania

	Intervention (***N*** = 336)	Control (***N*** = 336)	OR (95% CI)	***P*** value
	***n*** (%)	***n*** (%)		
Participants with *Ascaris lumbricoides* hand contamination	21 (6)	35 (10)	0.84 (0.391.81)	0.646
Participants with hookworm hand contamination	3 (1)	7 (2)	0.97 (0.165.77)	0.970
Hand contamination with coliforms	154 (46)	171 (51)	0.90 (0.551.46)	0.642
Moderate or high^a^ intensity hand contamination with coliform	61/154 (40)	95/171 (56)	0.67 (0.261.73)	0.451
Hand contamination with *Escherichia coli*	21 (6)	21 (6)	1.07 (0.452.55)	0.859
Moderate or high^a^ intensity hand contamination with *Escherichia* coli	5/21 (43)	12/21 (86)	0.74 (0.143.94)	0.727

^a^The quantification of total coliforms and *Escherichia coli* was categorised into mild (9 10^3^CFU/ml), moderate (1099 10^3^CFU/ml), and high (100 10^3^CFU/ml) intensities. Mild intensity infection used as reference for comparison of infection intensity

## Discussion

This trial assessed the potential effect of a hand hygiene intervention package on the prevalence and intensity of *Ascaris lumbricoides* and *Trichuris trichiura* infections among primary school children aged 6 to 12 years, 12 months after deworming using a single dose of albendazole. We had earlier shown that the intervention was well accepted by teachers, parents and children [23]. We found that the intervention resulted in increased reporting of handwashing with water and soap at school, but had failed to reduce the prevalence or intensity of any of the STH infections investigated. This might be due a number of reasons, including ineffectiveness of the handwashing intervention to reduce transmission in a school setting, occasional supply problems at schools with regard to the availability of water for handwashing, infections occurring in the home environment, alternative routes of STH transmission e.g. through contaminated food or drinking water or ingestion of soil, ineffectiveness of the MDA strategy with respect to *Trichuris trichiura* infection, and a lower than complete coverage of the MDA strategy due to absenteeism of some students. These results suggest that the education and hardware-based intervention to promote handwashing in schools and homes as delivered in this trial was not effective in reducing the burden of STH infections among school children when given in the context of routine deworming.

While the initial deworming successfully reduced the prevalence of *Ascaris lumbricoides* infection, it had no effect on *Trichuris trichiura* infections in spite of retreating remaining STH infections after the baseline survey. On the basis of these findings, we conclude that the MDA strategy currently used in Tanzania was not effective in reducing the burden of this infection. The lack of an effect of single dose albendazole treatment on *Trichuris trichiura* has been described by others [24]. We recommend the current MDA strategy for schools in Tanzania should be revised based on these findings.

There are several plausible explanations for the lack of observed effect of the handwashing intervention in our study. First, the handwashing intervention alone may not have been sufficient to reduce STH transmission within the school environment. While an association between handwashing promotion and STH infections has been observed in a range of observational and intervention studies [9, 10], in two recent trials from rural Kenya [25] and Bangladesh [26], handwashing intervention alone was not found to reduce STH infections. In contrast, a combined water improvement, sanitation and handwashing intervention package was shown to be effective [25, 26]. This suggests that hand hygiene interventions could contribute to the sustainable control of STH infections in settings of ongoing deworming programmes when provided as part of an integrated package, including water quality improvements and sanitation.

Second, our intervention may not have improved handwashing behaviours enough to provide an independent protective effect against STH transmission. Data on handwashing behaviour were based on self-reports and could be affected by desirability bias which may have differentially favoured intervention schools. To avoid this, we had originally planned to also systematically observe handwashing behaviour after children had visited the toilet. When this was piloted, we realised that the presence of the observer was akin to an intervention on its own and we abandoned this strategy. We can therefore not rule out that the intervention may have been weaker with regards to its effect on reported hygiene behaviour than our data suggest. The observed lack of an intervention effect on hand contamination with coliform bacteria and with *Escherichia coli* would lend support to this interpretation, although we saw a trend towards lower infection intensity in the intervention arm.

At schools, our intervention consisted of hygiene promotion intervention targeting children focused on three teacher-lead sessions incorporating hygiene education and games accompanied by new handwashing stations and other small-scale infrastructural changes. This intervention was deliberately designed to be replicable and scalable by the local education system without larger financial investments. The average investment and maintenance costs were GBP 88.2 per handwashing facility. The production was done by local craftsmen using locally available materials. Major repairs were not needed during the duration of the study. The compromise in intervention intensity made to promote scalability resulting in a lack of behavioural or health impact has been observed in other handwashing trials in domestic settings [27]. Specifically, the Mikono Safi intervention may not have been sufficient to allow for routine handwashing among children. A qualitative study of student and teacher participating in the Mikono Safi intervention found high rates of knowledge and strong motivation for handwashing among students [23]. However, this same study found that water and soap for use in handwashing were not consistently available to students, either due to periodic disruptions in water supply or because soap was not reliably available for students to use. Students in the intervention group may not have been able to wash hands frequently enough to interrupt pathogen transmission.

Third, other factors may have contributed to re-infection after deworming. While parents were given information on the transmission routes of helminth infections along with advice about actions they could take at home to protect children, changes in the domestic environment were modest and focused primarily on improving sanitation infrastructure (manuscript in development). The environment at home may, therefore, have been an important source of recontamination independent of any school-based improvements. We cannot rule out that other environmental transmission routes may have played a role: contaminated drinking water has been observed as a possible source of infection elsewhere [28], and this may have occurred also in our study given that the majority of households depended on unprotected springs and rivers which may pose a risk of STH infection. Contaminated food may also have contributed, in particular insufficiently cleaned vegetables or fruit [29, 30]. High proportion of students reported eating soil, and this may have contributed to helminth re-infection and episodes of diarrhoea. It is also possible that students from schools in the control arm in Bukoba town may have had contact with school mates from schools allocated to the intervention arm. This may have diluted any measurable intervention effect. However, in rural areas, the distance between communities were large and contamination nearly impossible, and because there was not even a trend suggesting an intervention effect, we feel that contamination is an unlikely explanation for the observed lack of effect.

A major strength of our study was its design, i.e. a cluster randomised trial with a large sample size. Another strength was that at the planning stage pre-trial data had been available on STH prevalence from 51 schools in the region. The co-efficient of variation could therefore be determined and inform sample size calculations, and schools with comparatively low STH prevalence excluded thereby increasing power of the study to detect a potential intervention effect. Furthermore, our handwashing intervention combined classical information giving, subconscious behaviour modification through environmental nudges and emotional engagement of parents and was therefore well-designed to trigger the desired behaviour change. Lastly, the long follow-up period of 12 months between deworming and end-line assessment allowed to study intervention effects beyond potentially short-lived behaviour modifications.

A major limitation of the study was its inability to causatively assess the potential role of the handwashing intervention on the transmission of *Trichuris trichiura* infection, due to the failure of single-dose treatment with 400 mg of albendazole to eliminate this specific STH. We informed the national NTD control programme, recommending a revision of the treatment regimen. Another limitation was that data on handwashing behaviour was based on self-reports and could be affected by desirability bias which may have differentially affected our results in the two trial arms. Furthermore, there was a slightly higher proportion of participants who did not provide stool samples at end-line in the control arm, and this could have introduced some selection bias. However, there was no evidence that collection of stool samples at end-line differed between intervention and control groups.

## Conclusions

In conclusion, the Mikono Safi handwashing intervention has been effective in fostering reported handwashing at school. While it was hoped that the intervention may also lead to an improvement of handwashing behaviour in the home, self-reports did not show such a secondary effect. Importantly, the intervention did not show the desired effect on maintaining the success of MDA on STH infection, probably due to infection occurring in the home environment and possibly due to other transmission routes which may have included contaminated water or food.

## Data Availability

De-identified participant data analysed in this report are available and can be assessed at an online data repository [31].
